# Efficient Tracing of the SARS-CoV-2 Omicron Variants in Santa Barbara County Using a Rapid Quantitative Reverse Transcription PCR Assay

**DOI:** 10.3390/diagnostics12112805

**Published:** 2022-11-15

**Authors:** Zach Aralis, Stewart Comer, Henning Ansorg, Carl Palmer, Jennifer Smith, Stuart C. Feinstein, Lynn N. Fitzgibbons, Carolina Arias

**Affiliations:** 1 Department of Molecular, Cellular, and Developmental Biology, University of California, Santa Barbara, CA 93106, USA; 2 Department of Biomolecular Science and Engineering, University of California, Santa Barbara, CA 93106, USA; 3 Santa Barbara County Public Health Department, Santa Barbara, CA 93106, USA; 4 Department of Pathology, Santa Barbara Cottage Hospital, Santa Barbara, CA 93106, USA; 5 Pacific Diagnostic Laboratories, Santa Barbara, CA 93106, USA; 6 LegacyWorks Group, Santa Barbara, CA 93106, USA; 7 California NanoSystems Institute, University of California, Santa Barbara, CA 93106, USA; 8 Neuroscience Research Institute, University of California, Santa Barbara, CA 93106, USA; 9 Department of Medical Education, Division of Infectious Diseases, Santa Barbara Cottage Hospital, Santa Barbara, CA 93106, USA; 10 Center for Stem Cell Biology and Engineering, University of California, Santa Barbara, CA 93106, USA; 11 Infectious Disease Initiative, Chan Zuckerberg BioHub, San Francisco, CA 93106, USA

**Keywords:** SARS-CoV-2, omicron, qPCR, public health, NGS, molecular assay, epidemiology

## Abstract

The emergence of the SARS-CoV-2 Omicron variant in 2021 is associated with a global surge of cases in late 2021 and early 2022. Identifying the introduction of novel SARS-CoV-2 variants to a population is imperative to inform decisions by clinicians and public health officials. Here, we describe a quantitative reverse transcription PCR-based assay (RT-qPCR) targeting unique mutations in the Omicron BA.1/BA1.1 and BA.2 viral genomes. This assay accurately and precisely detect the presence of these Omicron variants in patient samples in less than four hours. Using this assay, we tested 270 clinical samples and detected the introduction of Omicron BA.1/BA1.1 and BA.2 in the Santa Barbara County (SBC) population in December 2021 and February 2022, respectively. Identifying Omicron variants using this RT-qPCR assay showed complete concordance with whole viral genome sequencing; both assays indicated that Omicron was the dominant variant in SB County. Our data substantiate that RT-qPCR-based virus detection assays offer a fast and inexpensive alternative to NGS for virus variant-specific detection approach, which allows streamlining the detection of Omicron variants in patient samples.

## 1. Introduction

SARS-CoV-2, the causative agent of the COVID-19 pandemic, is a single strand positive RNA virus of the coronavirus family. COVID-19 has caused a devastating number of cases and deaths since it was officially declared a pandemic on 11 March 2020. Despite the development of successful vaccines and global vaccination efforts, SARS-CoV-2 continues to spread globally [1,2,3]. A challenge in controlling COVID-19 is the emergence of SARS-CoV-2 variants resulting from mutations that accumulate in the viral genome. While many of these mutations are of little to no consequence, others can provide a higher viral fitness by increasing virus transmission efficiency, conferring resistance to immune responses, and impacting the severity of the disease [4,5,6]. SARS-CoV-2 variants that pose a significant risk to the global community are designated Variants of Concern (VOC) by the World Health Organization (WHO) [7]. The control of SARS-CoV-2 transmission relies on our capacity to diagnose COVID-19 efficiently, detect the emergence of new viral lineages, and determine variant prevalence in the population [8,9,10,11].

One of the most infectious SARS-CoV-2 variants identified, Omicron BA.1 (originally B.1.1.529), was initially detected in South Africa in November of 2021, where it rapidly outcompeted other viral variants in the region [12]. In November 2021, the WHO classified SARS-CoV-2 Omicron as a VOC and linked it to a global upsurge of cases at the end of 2021 [13]. By December 2021, the highly transmissible Omicron variant had caused more than half of all daily SARS-CoV-2 infections [14]. Omicron carries numerous mutations, including 30 in the Spike (S) protein, which enhance binding to the cellular receptor ACE2 and increase immune evasion [15,16,17,18,19,20]. The BA.1.1 sublineage shows all the mutations found in BA.1 and the R346K substitution [21].

The high transmission rates of Omicron forecasted the emergence of sublineages, for example, BA.2, a highly contagious variant first detected in December 2021 and classified as a VOC in February 2022 [22]. While the global prevalence of BA.2 was lower than that of BA.1/BA.1.1 through January and February of 2022, it accounted for over 50% of the cases sequenced by the first week of March. Several mutations in the S protein distinguish BA.2 from BA.1/BA.1.1 and may enhance this variant’s transmissibility and immune evasion [22].

The numerous mutations in the S protein in BA.1/BA1.1 and BA.2 limit the options for treatment; of the three monoclonal antibody treatments approved for early use by the FDA, only Sotrovimab is effective against BA.1/BA.1.1 Omicron variant. However, this monoclonal antibody is ineffective in treating the BA.2 Omicron Variant [23]. Even though testing each patient to identify the infecting viral variant and tailor individual treatment is impractical, understanding the prevalence of specific variants in the community can inform decisions about using monoclonal antibodies and other COVID-19 therapies.

Next-generation sequencing (NGS) of the viral genome is the standard method to determine the SARS-CoV-2 variants in a sample. While NGS provides a wealth of information on the mutations present in an individual strain of the virus, it typically takes days to weeks to accurately identify viral variants. Rapid and straightforward methods to identify Omicron and other emerging viral variants are urgently needed to provide clinicians and public health officers with essential real-time information on the prevalence of specific SARS-CoV-2 variants in the population. In response to this emergency, several groups have developed alternative methods to detect viral variants in clinical samples (Table 1).

Here, we developed an RT-qPCR-based assay to distinguish between the Omicron BA.1/BA.1.1 and BA.2 variants of SARS-CoV-2 by targeting a set of variant-specific mutations in the S1 domain. We designed variant-specific RT-qPCR primers after honing in on viral genomic regions characterized by significant mutations, insertions, or deletions unique to specific variants. This strategy is versatile and can be easily adapted to identify emerging variants in the population. Using this simple RT-qPCR-based assay in residual diagnostic samples, we identified the BA.1/BA1.1 and BA.2 Omicron variants in patient samples with 100% accuracy compared to sample-matched NGS results. Our assay also provides a rapid, cost-effective alternative for SARS-CoV-2 variant identification; the time from assay design to deployment in patient samples is approximately one week, and its cost is lower than NGS-based approaches. Notably, the development of our assay was informed by the needs of our local public health department and hospitals. By immediately disseminating the results of our assays with health officials and clinicians, we provided vital information to treat active cases in the region and help manage the dramatic rise in COVID-19 cases in Santa Barbara County.

## 2. Materials and Methods

### 2.1. Primer Design

**Figure 1:**
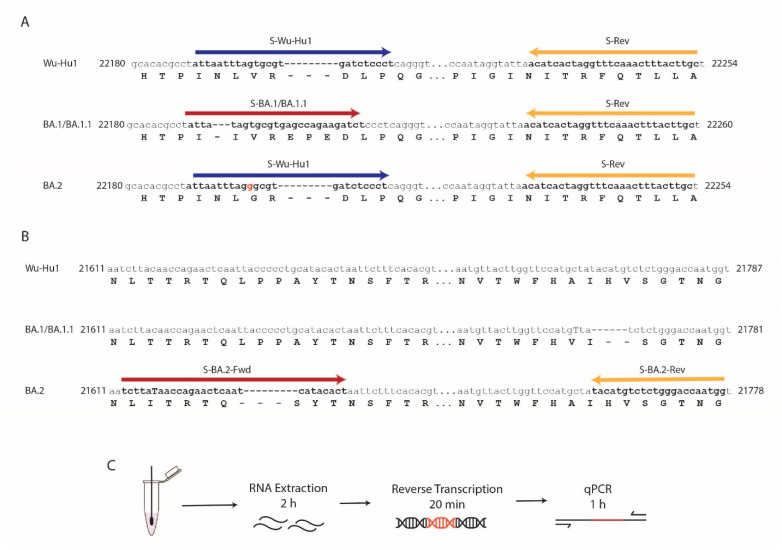
Primer Positions and Method overview. Genomic coordinates of the primer binding locations in the Wu-Hu1, BA.1/BA.1.1, and BA.2 viral variant genomes for (**A**) the BA.1/BA.1.1 targets and (**B**) the BA.2 targets. (**C**) Schematic and estimated timeline of RT-qPCR assay.

The coordinates of all primers and sequences are based on the SARS-CoV-2 Wuhan-Hu-1 (Wu-Hu1) genome (accession number NC_044512.2). The primers for BA.1/BA.1.1 specific targets were designed to target a 3-nucleotide deletion at position 22194-22196 and a 9-nucleotide insertion previously reported for this variant [33,34]. For BA.2, specific primers were designed to target a 9-nucleotide deletion at position 21633–21641 and the lack of a 6-nucleotide deletion at amino acids 69/70 present in BA.1/BA.1.1 and other VOCs [35]. The positions of the primers are illustrated in Figure 1, and the primer sequences are shown in Table 2.

### 2.2. Sample Collection and RNA Extraction

Clinical samples were acquired as residual NP swabs stored in Universal Transport Media (UTM) or Viral Transport Media (VTM). Samples were inactivated at 56 °C for 30 min, and RNA was extracted using the QIAamp MinElute Virus Spin Kit [Qiagen, 57704] from 140 µL of the sample, and eluted in 50 µL. The Santa Barbara Cottage Hospital IRB reviewed and approved pre-and post-analytical protocols. This study followed the Strengthening the Reporting of Observational Studies in Epidemiology (STROBE) reporting guideline for cohort studies.

### 2.3. RT-qPCR

Viral RNA was reverse transcribed by mixing 8 µL of extracted RNA with 2 µL of LunaScript RT SuperMix [NEB, Ipswich, MA, USA; E3010L], followed by incubation using the thermal profile: 25 °C for 2 min, 55 °C for 10 min, and 95 °C for 2 min. A qPCR master mix was prepared by combining 5 µL of nuclease-free water, 2 µL of 5µM S-Omicron or S-Wu-Hu1 Fwd primer, 2 µL of 5 µM S-reverse primer, 10 µL of the PowerUp™ SYBR™ Green Master Mix [Applied Biosystems, Waltham, MA, USA; A25741], and 1 µL of cDNA, for a total reaction volume of 20 µL. All components were gently mixed by pipetting, and the reactions were collected by centrifugation using a tabletop centrifuge. The reactions were run on a Bio-Rad CFX96 Touch using the following thermal protocol: 50 °C for 2 min; 95 °C for 2 min; 40 cycles of 95 °C for 15 s followed by 60 °C for 1 min, and the plate read in the SYBR/Fam channel. Data were analyzed using the Bio-Rad CFX Maestro software with a single threshold to determine the quantification cycle.

### 2.4. Data Interpretation

The assay was considered valid if the samples had a Ct value equal to or lower than 37 in the reactions with the S-BA.1/BA.1.1, BA.2 or S-Wu-Hu1 reaction. This cutoff is defined as the Ct value below by which virus variants in all samples could be successfully determined via NGS. Samples were defined as SARS-CoV-2 BA.1/BA.1.1 or BA.2 if the Ct value for the sample was equal to or lower than 37 in the qPCR reaction, including the S-Omicron BA.1/BA.1.1 or BA.2 Fwd primer. In the qPCR reaction, samples with Ct values above 37, including the S-Omicron or S-Wt primers, were deemed inconclusive. In limited cases, a small number of samples with low Ct values for one target had background amplification for one of the other targets, likely caused by non-specific amplification typically observed in mutation-specific qPCR assays [36,37,38]. In these cases, if the difference between Ct values was greater than 10 Ct, the target with the higher value was not considered (i.e., undetermined).

### 2.5. Amplicon Library Generation, Next Generation Sequencing, Phylogenetic Analysis

Viral cDNA was generated using LunaScript RT SuperMix, followed by incubation using the thermal profile: 25 °C for 2 min, 55 °C for 10 min, and 95 °C for 2 min. The cDNA was amplified using the SARS-CoV-2 genome tiling primer pools from the UCSF CAT COVID-19 Tailed 275bp ARTIC V3 Protocol [39]. The PCR reaction was prepared by mixing 5 µL of Q5 Hotstart 2X Master Mix [NEB, M0494S], 1.8µL of primer pool 1 or 2, 2.2 µL of nuclease-free water, and 1 µL of cDNA per sample. Amplification was carried out at 98 °C for 30 s, followed by 35 cycles of 95 °C for 15 s and 63 °C for 5 min. Pools 1 and 2 were combined, diluted 100-fold in nuclease-free water, and indexed using NEBNext dual index oligos for the Illumina [NEB, E6440S]. Equal sample volumes were pooled, cleaned up with AMPureXP beads, and sequenced on a NextSeq 500 instrument using a NextSeq 500/550 Mid Output Kit v2.5 (300 Cycles) kit. The demultiplexed FASTA files were uploaded to CZ ID for alignment. Consensus sequences were uploaded to Pangolin COVID-19 Lineage Assigner for variant identification. Consensus sequences generated by NGS were uploaded to Nexstrain for phylogenetic analysis [40]. Phylogenetic trees were visualized with the ggtree package in R [41].

## 3. Results

**Figure 2:**
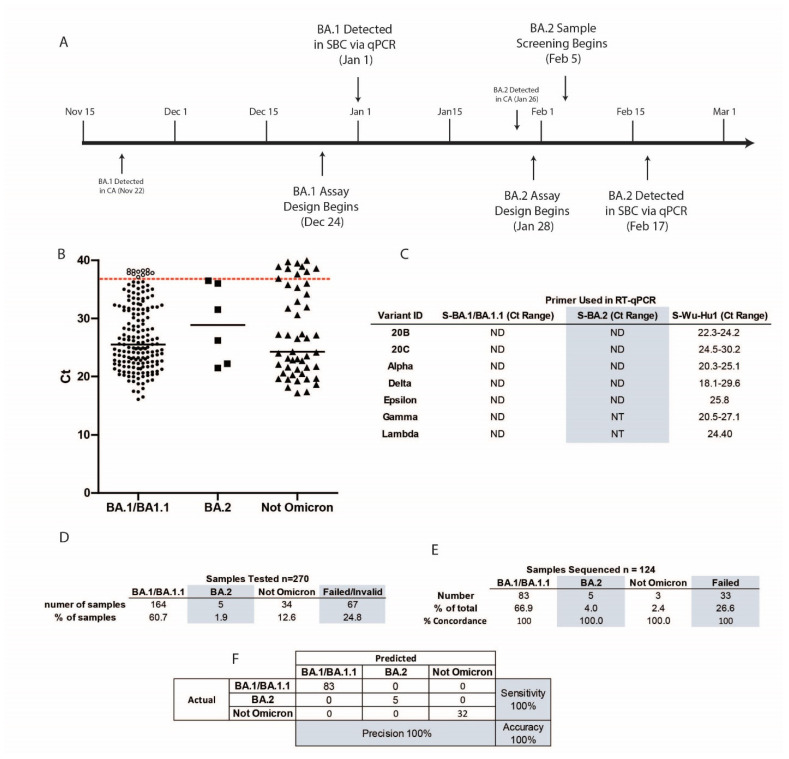
Design timeline and metrics of the Omicron variant-specific RT-qPCR. (**A**) Timeline of events around the design, optimization, and implementation of the Omicron variant-specific RT-qPCR in Santa Barbara County. (**B**) Ct values for all samples tested. The red dashed line indicates the cutoff at a Ct of 37. (**C**) Ct values of a panel of samples known to be variants other than Omicron BA.1, BA.1.1, and BA.2. (**D**) Results of all 270 samples tested. (**E**) Percent concordance and (**F**) confusion matrix of samples with whole viral NGS and variant-specific RT-qPCR data. ND: not detected; NT: not tested.

The Omicron variant of SARS-CoV-2 initially reported at the end of November 2021 in South Africa, is characterized by numerous mutations throughout the genome. Most of these mutations, 30 in BA.1/BA.1.1 and 20 in BA.2, have been described in the gene encoding the Spike (S) protein gene. To develop a quantitative reverse transcription PCR (RT-qPCR)-based assay to precisely detect the Omicron variant sublineages BA.1/BA.1.1 and BA.2 in clinical samples, we designed primers targeting unique mutations present in the S gene of each of these variants. The BA.1/BA.1.1 primers target the N211 deletion (N211del), the L212I substitution, and the 214 EPE insertion (ins214EPE) (Figure 1A). The BA.2 primers target the T19I substitution and L24/P25/P26 deletion (Figure 1B). In parallel, we designed the S-Wu-Hu1 primer targeting the region in the S gene encoding for amino acids (AA) 210–217 that would recognize all SARS-CoV-2 variants of concern, except for Omicron BA.1/BA.1.1 (Figure 1A). Our RT-qPCR assay takes less than 4 h from RNA extraction to readout (Figure 1C). We successfully designed and validated the assay and obtained results from patient samples within one week (Figure 2A).

To validate this assay, we obtained 270 residual SARS-CoV-2 positive nasopharyngeal swab samples collected for diagnostic purposes between December 2021 and February 2022 with undetermined viral variants (Figure 2A. Timeline). As a control, we used 29 retrospective residual SARS-CoV-2 positive nasopharyngeal swab samples we previously identified by viral genome sequencing as 20B, 20C, Epsilon (CAL.20C), Gamma (P.1), Lambda (C.37), Alpha (B.1.1.7), and Delta (B.1.617.2 and AY) (Figure 2B). Using our RT-qPCR assay in this cohort, we detected BA.1/BA.1.1 in 164 samples (60.7% of total), BA.2 in 5 samples (1.9% of total), and other variants in 34 samples (12.6% of total). Our results confirmed the overwhelming presence of SARS-CoV-2 Omicron in most samples collected in late 2021/early 2022. The 67 remaining samples failed due to a lack of amplification and detection by RT-qPCR with the S-Omicron or the S-Wu-Hu1 primers, possibly due to low viral load or poor sample preservation (Figure 2D). The control samples previously identified to contain viral RNA for the SARS-CoV-2 variants 20B, 20C, Alpha, Delta, Epsilon, Gamma, and Lambda were all positive by RT-qPCR with the S-Wu-Hu1 but not with the S-Omicron BA.1/BA.1.1 specific primers (Figure 2C).

**Figure 3:**
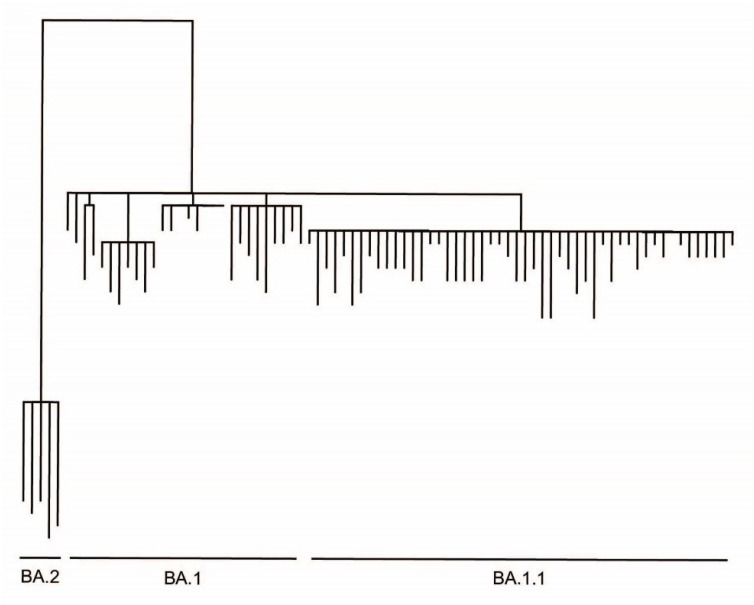
Phylogenetic analysis based on whole-genome sequences of SARS-CoV-2 Omicron variants detected in Santa Barbara County.

**Figure 4:**
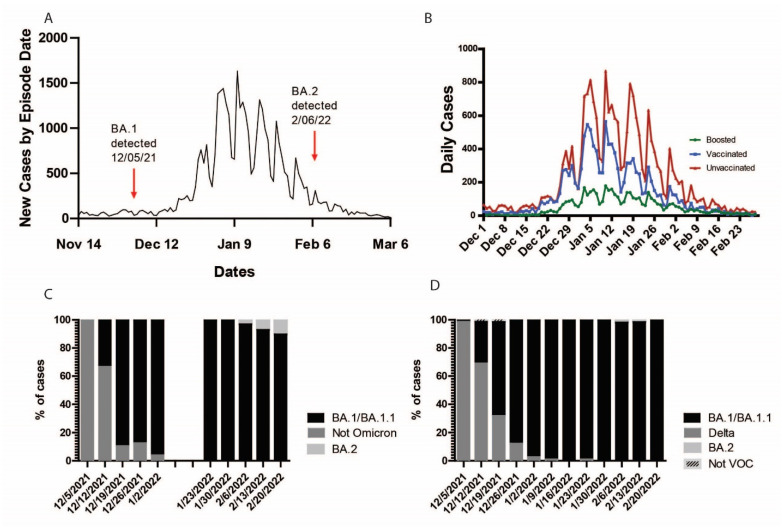
Daily cases and relative variant proportions in Santa Barbara County. (**A**) Total new COVID-19 cases by episode date and (**B**) new cases in boosted, vaccinated, and unvaccinated individuals in Santa Barbara County reported by the SBCPHD [42]. (**C**) Relative proportion of omicron samples in Santa Barbara County, determined via variant-specific RT-qPCR and (**D**) whole viral NGS.

To confirm the presence of the Omicron variant in these clinical specimens and evaluate the accuracy of our assay, we sequenced the viral genome in 124 of the 270 patient samples that were tested using our RT-qPCR assay. We identified 83 SARS-CoV-2 Omicron BA.1/BA.1.1 (91.2% of samples successfully sequenced), 5 Omicron BA.2 (5.5%), and 3 SARS-CoV-2 Delta (3.3%) (Figure 2E). Thirty-three samples failed viral genome sequencing, likely due to low viral load or poor sample preservation. The identification of the SARS-CoV-2 variants using whole-genome sequencing showed one hundred percent concordance with our RT-qPCR assay identifications, highlighting the specificity and accuracy of our assay (Figure 2F). Genetic and phylogenetic analyses of the sequenced genomes show defining mutations of the omicron sublineages and the presence of three distinct clusters corresponding to BA.1, BA.1.1, and BA.2 (Figure 3). The sublineages BA.1 and BA.1.1 were introduced late in 2021 and continued circulating in the Santa Barbara County (SBC) population throughout early 2022. We detected the introduction of BA.2 in the 6th week of 2022, with sustained transmission until the end of our sampling period (Figure 4A,C). Our phylogenetic analyses support the local transmission of these variants (Figure 3).

The presence of Omicron (BA.1, BA.1.1, and BA.2) in the clinical samples from SBC is linked to a dramatic surge in the number of cases since December of 2021, mainly in the unvaccinated population (Figure 4A,B). The weekly distribution of SARS-CoV-2 variants in clinical samples, determined by RT-qPCR (Figure 4C) or whole genome sequencing (Figure 4D), shows the dominance of the Delta variant in SBC throughout November and the initial detection of Omicron in the week of 5 December 2021. The rapid expansion of the Omicron variant in the population is seen in the following two weeks, with the complete replacement of the Delta variant and the dominance of Omicron found present in 100% of the samples tested by the first week of January 2022 (Figure 4C). We detected the BA.2 sublineage during the second week of February 2022, with low prevalence (below 5%) throughout the rest of the month.

## 4. Discussion

As new SARS-CoV-2 variants emerge and fuel COVID-19 cases worldwide, it is critical to continue developing tools for the rapid identification and characterization of viral variants that will inform clinical and public health decisions. In SBC, our early adoption of next-generation sequencing methods for genomic surveillance of SARS-CoV-2 became a fundamental part of the local COVID-19 pandemic response. NGS of viral genomes shed light on the regional distribution and prevalence of viral variants and informed prevention and clinical intervention strategies [43]. While informative and critical for early and guided public health responses, state-wide and local next-generation surveillance initiatives provided results with a one to six weeks delay. Omicron’s rapid emergence and high transmissibility presented a precipitous local surge risk, as seen in other communities [13]. Thus the successful control of potential Omicron outbreaks required the prompt detection of this variant in patient samples. From the clinical management perspective, a priori knowledge of the presence and prevalence of Omicron variants in the patient population can help guide the selection and use of limited-supply monoclonal antibody therapies [20].

Many groups around the world have responded to the emergence of these highly infectious variants by developing several assays to detect viral variants of concern [24,25,26,27,28,29,30,31,32]. In this study, we collaborated with SBC public health officers and clinicians to rapidly develop and implement a simple and highly specific RT-qPCR assay to detect Omicron variants in residual diagnostic clinical samples. The primers we use in this RT-qPCR assay target unique combinations of mutations in the BA.1/BA.1.1 and BA.2 viral genomes, which differentiate this assay from others reported in the literature (see Table 1). With this design, PCR amplification will only occur when the primer binds the cDNA derived from the target viral variants, resulting in the accurate identification of specific viral sublineages. This rapid assay (4 h from start to completion) requires basic equipment and reagents commonly found in molecular biology and clinical laboratories, is scalable, and demands minimal optimization.

This simple RT-qPCR-based assay allowed us to rapidly identify the introduction and rapid dominance of Omicron BA.1/BA.1.1 and BA.2 in the SBC community. These valuable data, informed clinicians and discouraged the use of monoclonal antibodies to manage active cases. Moreover, identifying Omicron in the SBC population triggered enhanced testing and contact tracing by the SB Department of Public Health to control viral transmission. The success and rapid identification of BA.1/1.1 and BA.2 in patient samples highlight this assay’s straightforward design and adaptability. However, the usability of this RT-qPCR-based assay for detecting other emerging viral variants will require careful selection of the amplification regions. The design of primers to achieve specific amplification may be challenging, particularly to differentiate highly similar variants, for example BA.2 and the recently identified BA.4 and BA.5 sublineages [44].

As the COVID-19 pandemic evolves, rapidly deployable variant detection methods will remain indispensable to enhance public health. Our results demonstrate that a simple assay, needing minimal troubleshooting and optimization, successfully captured the introduction of SARS-CoV-2 variants in the population and provided valuable real-time data during emerging surges to guide clinical and public health efforts. Most importantly, this study illustrates the power and impact of collaborative work and open communication between academic research laboratories, clinicians at local hospitals, and public health officers to rapidly respond to a public health emergency, significantly improving outbreak control responses and public safety.

## Figures and Tables

**Table 1:** Current assays for SARS-CoV-2 variant detection/identification.

Method	SARS-CoV-2 Variants	Mutations Targeted	Reference
RT-qPCR	Omicron	N:S135R; N:I189V; S:A27S; S:S371L; N:F108L; S:G446S; S:T547K; S:L981F; N:T24I; N:L1266I; S:V213G; S:R408S	Li et al. [24]
RT-qPCR	Omicron	N:31Del; N:32Del; N:33Del; N:P13L; N:R203K; N:G204R	Ippoliti et al. [25]
RT-qPCR	Omicron	S:E484A; S:Y505H	Corbisier et al. [26]
RT-ddPCR	Omicron	S:S477N; S:T478K; S:E484A; S:G496S; S:Q498R; S:N501Y S:Y505H	Mills et al. [27]
High-Resolution Melting analysis	Omicron	S:G339D; S:N440K; S:G446S; S:D796Y	Koshikawa et al. [28]
High-Resolution Melting analysis	BA1.1/BA1.2	S:R408S; S:G446R; S:447N; S:T448K	Aoki et al. [29]
MeltaArray	Alpha, Delta, BA.1, BA.2, BA.3, and BA.4/5	S:A67V; S:T95I; S:Del69/70; S:G142D; S:Del143/145; S:N211I; S:Del212; S:Ins214EPE; S:G339D; S:S371L; S:S373P; S:S375F; S:K417N; S:N440K; S:G446S; S:S477N; S:T478K; S:E484A; S:Q493R; S:G496S; S:Q498R; S:N501Y; S:Y505H; S:T547K; S:D614G; S:H655Y; S:N679K; S:P681H; S:N764K; S:D796Y; S:N856K; S:Q954H; S:N969K; S:L981F;	Yan et al. [30]
Multiplex Fragment Analysis	Delta, Mu, Lambda, Omicron	S:Del69/70; S:Ins146N; S:Del144; S:Del156/157; S:Del143/145; S:Del241/243; S:Ins214EPE; S:Del211; S:Del247/253; S:L452R S:E484K; S:N501Y; ORF1A:Del3675/3677; ORF8:Ins28269/28273; ORF8:Del119/120; ORF8:Del31/33;	Clark et al. [31]
Mass Spectrometry	Iota, Alpha, Delta, and Omicron	S:L5F; S:S13I; S:L18F; S:T19R; S:Del69/70; S:D80A; S:D80G; S:T95I; S:Del144; S:W152C; S:D215G; S:Del242/244; S:D253G; S:K417N; S:K417T; S:N439K; S:L452R; S:Y453F; S:S477N; S:T478K; S:E484Q; S:E484K; S:Q493K; S:N501Y; S:N501T; S:A570D; S:D614G; S:Q677H; S:Q677P; S:P681H; S:P681R; S:I692V;	Hernandez et al. [32]

**Table 2:** Primer sequences used in this study.

Name	Bases (5′–3′)	Target
S-BA.1/BA.1.1 Fwd	ATTATAGTGCGTGAGCCAGAAGATCT	BA.1/BA.1.1
S-Wu-Hu1 Fwd	ATTAATTTAGTGCGTGATCTCCCT	All Other variants
S-Rev	GCAAGTAAAGTTTGAAACCTAGTGATGT	All Variants
S-BA.2 Fwd	TCTTATAACCAGAACTCAATCATACACT	BA.2
S-BA.2 Rev	CCATTGGTCCCAGAGACATGTA	BA.2

## Data Availability

The data presented in this study are available on request from the corresponding author.
